# MICROBIAL PATTERNS AND DRUG SENSITIVITY TEST OF BACTERIAL AND FUNGAL INFECTION IN COVID-19 PATIENTS AT THE NATIONAL REFERRAL HOSPITAL IN NORTH SUMATRA, INDONESIA

**DOI:** 10.21010/Ajidv19i1.5

**Published:** 2024-10-25

**Authors:** SIBARANI Ursula Regina Florense, SINAGA Bintang Yinke Magdalena, Muntasir, MUTIARA Erna

**Affiliations:** 1 Department of Pulmonology and Respiratory Medicine, Faculty of Medicine, Universitas Sumatera Utara, Medan, Indonesia; 2 Department of Pulmonology and Respiratory Medicine, Adam Malik Hospital, Medan, Indonesia; 3 Faculty of Public Health, Universitas Sumatera Utara, Medan, Indonesia

**Keywords:** COVID-19, bacterial, fungal, antibiotics, antifungal

## Abstract

**Background:**

Bacteria or fungi in COVID-19 involves several mechanisms that can affect immune system and also can increase severity of symptoms. The incidence of bacterial or fungal infections is common in patients with viral respiratory tract infections. The aim of this study was to determine the microbial patterns and sensitivity tests of bacterial and fungal infection in COVID-19 patients at National Referral Hospital in North Sumatra, Indonesia.

**Materials and Methods:**

A cross-sectional design, where data was obtained from a 100 COVID-19 patients medical records, from December 2020 to April 2021. This study employed total sampling, those that fit the inclusion and exclusion criteria.

**Results:**

A total population of 100 COVID-19 patients were included, with bacterial infections in 26 patients (26%), fungi in 5 patients (5%), bacteria and fungi in 5 patients (5%). The most common bacterial pathogen was *Acinetobacter baumannii* in 14 patients (45.1%), followed with *Klebsiella pneumonia* in 5 patients (16.1%), *Pseudomonas aeruginosa* in 5 patients (16.1%) and *Escherichia coli* in 3 patients (9.6%). The most sensitive antibiotic in *Acinetobacter baumannii* was Amikacin (57.14%). The most common fungal infection was *Candida albicans* in 5 patients (50.0%) and mostly sensitive to Fluconazole, Voriconazole, Caspofungin, Amphotericin B, Micafungin and Flucytosine (60.0%).

**Conclusion:**

Bacterial and fungal infections can occur in COVID-19 patients, bacterial infection most commonly found in this study. The most sensitive antibiotic or anti-fungal is different for each bacterial or fungal found, and can be used as a basis for antibiotic selection for COVID-19 patients.

## Introduction

On March 11, 2020, the World Health Organization (WHO) declared the coronavirus disease of 2019 (COVID-19) a global pandemic. As of the latest update on June 19, 2023, in Indonesia, there were 6,811,989 confirmed cases positive for COVID-19, resulting in 161,871 deaths. (Indonesian Health Ministry, 2024). Bacterial and fungal infections have been reported in COVID-19 patients, although there is still a lack of information regarding the incidence and prevalence. The occurrence of infections caused by other pathogens such as bacteria or fungi in COVID-19 involves several mechanisms that can affect immune system and provide opportunities for infection by additional pathogens. (Chen X *et al.*, 2020) The incidence of infection due to other pathogens such as bacteria or fungi is very likely in patients infected with COVID-19 and will lead to increased severity of symptoms, and this can be affected by age, gender, COVID-19 severity, comorbid, history of antibiotic use, vaccination history and ventilator use. (Wu G *et al.*, 2023) A retrospective case study involving 55 patients with confirmed severe/critical COVID-19 and 166 patients with confirmed mild/moderate COVID-19, reported that the incidence of bacterial infection was 7.7% and the incidence of fungal infection was 3.2%. (Chen X *et al.*, 2020) Another retrospective study in Wuhan, China, found that the most common bacterial co-infections in COVID-19 patients were caused by *Acinetobacter baumannii* and *Klebsiella pneumonia*, while the most common fungal infections were caused by *Aspergillus flavus*, *Candida glabrata* and *Candida albicans*. (Chen N *et al.*, 2020)

Bacterial or fungal infections are known as a complication of viral respiratory infections. The pathophysiological mechanism of bacterial or fungal infection is respiratory epithelial damage where Severe Acute Respiratory Syndrome Coronavirus 2 (SARS-CoV-2), the virus that causes COVID-19, attacks respiratory epithelial cells and damages the integrity of the epithelial layer. This can weaken the natural defenses of the respiratory tract and facilitate the invasion of additional pathogens. SARS-CoV-2 can also disrupt or damage mucociliary defense mechanisms that are important in maintaining airway hygiene. This can lead to accumulation of mucus and increase the risk of bacterial or fungal infection. (Feldman C & Anderson R, 2021)

There is study about risk factors of bacterial infection in COVID-19. Sex, comorbidities, mechanical ventilation, invasive devices, combination of antibiotics, and glucocorticoid treatment were found to be related to other pathogen infections. Critical patients with invasive therapy and previous antibiotics use should be cautious with secondary bacterial infections. Patients who use ventilators have a greater potential for bacterial and fungal infections. (Wu G *et al.*, 2023) Empiric antibiotic administration must be adjusted to microbiological patterns and local resistance patterns in hospitals, considering there is potential risk for colonization of multi-resistant bacteria by prior antibiotic exposure, the use of antibacterials in patients with COVID-19 should be cautious. (Lee J *et al.*, 2023) The aim of this study was to determine the microbial patterns and sensitivity tests of bacterial and fungal infection in COVID-19 patients at Adam Malik Hospital, Medan, Indonesia.

## Materials and Methods

### Study design, setting and sampling

A retrospective cross-sectional study design was conducted at Adam Malik Hospital by observing data from the hospital’s medical records for 4 months, from December 2020 to April 2021. This study employed total sampling, those that fit the inclusion and exclusion criteria.

### Ethical consideration

Ethical clearance for this research was approved by Universitas Sumatera Utara Ethics Committee on August 14, 2023 No: 790/KEPK/USU/2023.

Of the 474 COVID-19 patients during the study period, 100 COVID-19 patients were tested for sputum culture. This study took patients data that fit the inclusion criteria, which were patients who confirmed for COVID-19 by reverse transcription polymerase chain reaction (RT-PCR) examination, aged over 18 years old, underwent sputum culture and sensitivity tests. The exclusion criteria are data or medical records that are incomplete. The RT-PCR involves ribonucleic acid (RNA) extraction, reverse transcription to convert RNA to complementary DNA (cDNA), PCR amplification and detection of amplified deoxyribonucleic acid (DNA) using fluorescent dyes, with temperature settings denaturation(95^o^C), annealing(55^o^C), and extension((72^o^C).

### Data collection

COVID-19 patient’s data were obtained from medical record and in accordance with inclusion and exclusion criteria. From the data obtained, data on COVID-19 patients who performed sputum culture were sought. Then we collect the data including age, gender, COVID-19 severity, comorbidities, growth of other pathogen, antibiotic history, vaccination history and history of ventilator used. Sputum culture results of COVID-19 patients were recorded based on the presence or absence of bacterial and or fungal growth, then the type of bacterial and or fungal and the results of antibiotic sensitivity tests for bacteria and anti-fungal sensitivity test for fungal.

## Data analysis

All data obtained were edited using the Excel program (Microsoft, Washington, USA). Data was grouped and examined by coding formulations for efficiency, resulting in all data presented in the form of frequency distributions and percentages.

## Results

### A. Patients characteristics

A total of 100 COVID-19 patients were included in this study, as presented in Table 1.

**Table 1:** Characteristics of the patients

Characteristics	n (%)
**Age (years)**	
<45	34 (34)
>45	66 (66)
**Gender**	
Female	41 (41)
Male	59 (59)
**COVID-19 severity**	
Moderate	66 (66)
Severe/critical	34 (34)
**Comorbidities**	
Yes	61 (61)
None	39 (39)
**Growth of other pathogen**	
Yes	36 (36)
None	64 (64)
**History of antibiotics**	
Yes	84 (84)
None	16 (16)
**History of COVID-19 vaccination**	
Yes	75 (75)
None	25 (25)
**History of ventilator used**	
Yes	2 (2)
None	98 (98)

The majority of patients were male (59%) and aged above 45 (66%). Based on the severity, 66% of the patients had moderate COVID-19, and 61% of the patients had comorbidities. Among all of the patients, organism growth was only detected in 36 patients. Most patients had a history of antibiotics (84%), had a COVID-19 vaccination (75%), and had no use of ventilators (98%).

### B. Frequency distribution of bacterial and fungal growth in COVID-19 patients

It was observed that 26 patients had bacterial infections, 5 patients had fungal infections, and 5 other patients had both bacterial and fungal infections (Table 2).

**Table 2:** Frequency distribution of COVID-19 patients with bacterial and fungal growth

Culture results	n	%
Bacteria	26	26
Fungi	5	5
Bacteria and fungi	5	5

Gram-negative bacteria were dominant found among the COVID-19 patients, including *Acinetobacter baumannii* (46.7%), *Klebsiella pneumonia* (16.7%), *Pseudomonas aeruginosa* (16.7%), *Escherichia coli* (10%), *Enterobacter cloacae* (3.3%), *Stenotrophomonas maltophilia* (3.3%), *Pseudomonas fluorescens (3.3%)*. While *Corynebacterium striatum* was the only gram-positive bacteria found (Table 3).

**Table 3:** Bacteria types in COVID-19 patients

Bacteria type	Bacteria species	n	(%)
Gram-negative (n=30)	*Acinetobacter baumannii* *Klebsiella pneumonia*	14 5	46.7 16.7
	*Pseudomonas aeroginosa*	5	16.7
	*Eschericia coli*	3	10
	*Enterobacter cloacae*	1	3.3
	*Stenotrophamonas maltophilia*	1	3.3
	*Pseudomonas fluorescens*	1	3,3
Gram-positive (n=1)	*Corynebacterium striatum*	1	100

The most common fungal growth among the COVID-19 patients with coinfection was *Candida albicans* (50%), followed by *Candida glabrata* (30%) and *Candida tropicalis* (20%) (Table 4).

**Table 4:** Fungal types in COVID-19 patients

Fungal species	n	(%)
*Candida albicans*	5	50
*Candida glabrata*	3	30
*Candida tropicalis*	2	20

### C. Antibiotic and antifungal sensitivity test in COVID-19 patients

Amikacin (57.1%) was the most sensitive antibiotic to *Acinetobacter baumannii*, followed by trimethoprim (40.0%), however, it was resistant (78.57%) to ceftazidime, ceftriaxone, ciprofloxacin, gentamicin and piperacillin/tazobactam, and it was intermediate to tigecycline (14.2) and ceftriaxone (7,2). *Pseudomonas aeruginosa* was sensitive to amikacin (100%) and gentamicin (100%), and was found to be resistant to tigecycline (100%). *Klebsiella pneumoniae* was highly susceptible to amikacin (100%), ertapenem (80%) and meropenem (80%). Although it showed resistance to ampicillin (100%), aztreonam (100%), ceftriaxone (80%), ciprofloxacin (80%), ampicillin/sulbactam (60%), gentamycin (60%), and trimethoprim (60%), and it was found intermediate to ampicillin sulbactam and ceftazidime (20%). Meropenem and tigecycline had 100% sensitivity to *Escherichia coli* and was 100% resistant to ceftriaxone and ciprofloxacin. *Stenotrophomonas maltophilia* was sensitive to trimethoprim and levofloxacin (100%) and resistant to ceftazidime (100%). Pseudomonas fluorescens was sensitive to amikacin, gentamycin, meropenem, levofloxacin (100%) and resistant to ceftazidime, ceftriaxone, ciprofloxacin, tigecycline and trimethoprim (100%). *Enterobacter cloacae* was sensitive to amikacin (100%), meropenem (100%) and piperacillin/tazobactam (100%) and was found to be resistant to ampicillin, ampicillin/sulbactam, ceftazidime, ceftriaxone, ertapenem, cefepime, ciprofloxacin, gentamycin, trimethoprim, and aztreonam (100%) and was found intermediate to piperacillin/tazobactam (100%) (Table 5).

**Table 5:** Antibiotics sensitivity to gram-negative in COVID-19 patients

Antibiotics	*Acinetobacter baumanii (n=14)*					

	Sensitive		Resistance		Intermediate	

	n	%	n	%	n	%
Amikacin	8	57.14	6	42.86	0	0
Ampicillin	0	0	3	21.42	0	0
Ampicillin/sulbactam	4	28.57	7	50.00	0	0
Ceftazidime	2	14.29	11	78.57	0	0
Ceftriaxone	0	0	11	78.57	2	14.2
Ertapenem	0	0	1	7.14	0	0
Cefepim	2	14.29	9	64.29	0	0
Ciprofloxacin	2	14.29	11	78.57	0	0
Gentamycin	2	14.29	11	78.57	0	0
Meropenem	3	21.42	10	71.43	0	0
Piperacillin/tazobactam	1	7.14	11	78.57	0	0
Tigecycline	5	5.71	1	7.14	2	14.2
Trimethoprim	6	42.86	6	42.86	0	0
Levofloxacin	0	0	2	14.29	0	0

**Table:** 

Antibiotics	*Pseudomonas aeruginosa (n=5)*					

	Sensitive		Resistance		Intermediate	

	n	%	n	%	n	%
Amikasin	5	100	0	0	0	0
Ceftazidime	4	80	1	20	0	0
Ceftriaxone	0	0	1	20	0	0
Cefepim	4	80	0	0	0	0
Ciprofloxacin	4	80	1	20	0	0
Gentamycin	5	100	0	0	0	0
Meropenem	4	80	1	20	0	0
Piperacillin/tazobactam	4	80	0	0	0	0
Tigecycline	0	0	5	100	0	0
Trimethoprim	0	0	1	20	0	0
Levofloxacin	3	60	0	0	0	0
Aztreonam	4	80	0	0	0	0

**Table:** 

Antibiotics	*Klebsiella pneumonia (n=5)*					

	Sensitive		Resistance		Intermediate	

	n	%	n	%	n	%
Amikacin	5	100	0	0	0	0
Ampicillin	0	0	5	100	0	0
Ampicillin/sulbactam	1	20	3	60	1	20
Ceftazidime	2	40	2	40	1	20
Ceftriaxone	1	20	1	80	0	0
Ertapenem	4	80	1	20	0	0
Cefepim	3	60	1	20	0	0
Ciprofloxacin	2	20	4	80	0	0
Gentamycin	1	40	3	60	0	0
Meropenem	4	80	1	20	0	0
Piperacillin/tazobactam	2	40	1	20	0	0
Tigecycline	3	60	1	20	0	0
Trimethoprim	1	20	3	60	0	0

**Table:** 

Antibiotics	*Eschericia coli (n=3)*					

	Sensitive		Resistance		Intermediate	

	n	%	n	%	n	%
Amikacin	2	66.67	0	0	0	0
Ampicillin	0	0	3	100	0	0
Ampicillin/sulbactam	2	66.67	1	33.33	0	0
Ceftazidime	1	33.33	2	66.67	0	0
Ceftriaxone	0	0	3	100	0	0
Ertapenem	3	100	0	0	0	0
Cefepim	2	66.67	1	33.33	0	0
Ciprofloxacin	0	0	3	100	0	0
Gentamycin	2	66.67	1	33.33	0	0
Meropenem	3	100	0	0	0	0
Piperacillin/tazobactam	1	33.33	1	33.33	0	0
Tigecycline	3	100	0	0	0	0
Trimethoprim	1	33.33	2	66.67	0	0

**Table:** 

Antibiotics	*Stenotrophomonas maltophilia (n=1)*					

	Sensitive		Resistance		Intermediate	

	n	%	n	%	n	%
Ceftazidime	0	0	1	100	0	0
Trimethoprim	1	100	0	0	0	0
Levofloxacin	1	100	0	0	0	0

**Table:** 

Antibiotics	*Pseudomonas fluorescens (n=1)*					

	Sensitive		Resistance		Intermediate	

	n	%	n	%	n	%
Amikacin	1	100	0	0	0	0
Ceftazidime	0	0	1	100	0	0
Ceftriaxone	0	0	1	100	0	0
Ciprofloxacin	0	0	1	100	0	0
Gentamycin	1	100	0	0	0	0
Meropenem	1	100	0	0	0	0
Tygecycline	0	0	1	100	0	0
Trimethoprime	0	0	1	100	0	0
Levofloxacine	1	100	0	0	0	0

**Table:** 

Antibiotics	*Enterobacter cloace (n=1)*					

	Sensitive		Resistance		Intermediate	

	n	%	n	%	n	%
Amikacin	1	100	0	0	0	0
Ampicillin	0	0	1	100	0	0
Ampicillin/sulbactam	0	0	1	100	0	0
Ceftazidime	0	0	1	100	0	0
Ceftriaxone	0	0	1	100	0	0
Ertapenem	0	0	1	100	0	0
Cefepim	0	0	1	100	0	0
Ciprofloxacin	0	0	1	100	0	0
Gentamycin	0	0	1	100	0	0
Meropenem	1	100	0	0	0	0
Piperacillin/tazobactam	1	100	0	0	1	100
Trimethoprim	0	0	1	100	0	0
Aztreonam	0	0	1	100	0	0

There is no antibiotic are sensitive to Corynebacterium striatum. Ciprofloxacin and piperacillin/tazobactam were resistance to *Corynebacterium striatum* (100%) (Table 6).

**Table 6:** Antibiotics sensitivity to gram-positive in COVID-19 patients

Antibiotics	*Corynebacterium striatum (n=1)*					

	Sensitive		Resistance		Intermediate	

	n	%	n	%	n	%
Ciprofloxacin	0	0	1	100	0	0
Meropenem	0	0	1	100	0	0
Levofloxacin	0	0	1	100	0	0

Fluconazole, voriconazole, caspofungin, amphotericin B, micafungin and flucytosine were all 60% sensitive to *Candida albicans*. *Candida glabrata* was sensitive to voriconazole, amphotericin B, micafungin and flucytosine (100%). *Candida tropicalis* was sensitive to all antifungal drugs tested (Table 7).

**Table 7:** Antifungal sensitivity in COVID-19 patients

Antifungal	*Candida albicans* (n=5)				*Candida glabrata* (n=3)				*Candida tropicalis* (n=2)			

	Sensitive		Resistance		Sensitive		Resistance		Sensitive		Resistance	

	n	%	n	%	n	%	n	%	n	%	N	%
Fluconazole	3	60	0	0	0	0	0	0	2	100	0	0
Voriconazole	3	60	0	0	3	100	0	0	2	100	0	0
Caspofungin	3	60	0	0	2	66.67	1	33.3	2	100	0	0
Amphotericine B	3	60	0	0	3	100	0	0	2	100	0	0
Micafungin	3	60	0	0	3	100	0	0	2	100	0	0
Flucytosine	3	60	0	0	3	100	0	0	2	100	0	0

## Discussion

Out of 100 patients who underwent culture examination, 36 patients (36%) with growth of bacterial, fungal or both. The frequency of bacterial and fungal infection in COVID-19 patients, 26 patients (26%) with bacterial infections, 5 patients (5%) with fungal infections, and 5 patients (5%) with mixed bacterial and fungal infections. The number of bacterial, fungal, or bacterial and fungal infections confirmed microbiologically from culture examination in COVID-19 patients is less than no bacterial or fungal growth 64 patients (64%). Respiratory tract infections caused by viruses, in this case SARS-CoV-2, often coexist with bacterial and fungal infections and often associated with mortality of patients. (Dimeglio C *et al.*, 2021) It is very important to understand a COVID-19 patient with bacterial infection which will greatly assist in proper antibiotics used and minimize negative consequences or excessive use of antibiotics. (Lee J *et al.*, 2023)

A rapid review study report that 6-15% of hospitalized COVID-19 patients are found to have a bacterial infection. From that report, the involvement of bacterial infection in SARS-CoV-2 infection and mortality is necessary to elucidate the causal relationship, which is crucial for effective treatment strategies in COVID-19 patients. (Farrell JM *et al.*, 2021) Another study also found from a total 74 bacterial infections, 7 fungal infections, and 7 viral infections where co-infection (31/989, 3.1%) and a total of 51 bacterial superinfections. Co-infection is defined if the diagnosis is made at the time or within the first 24 hours after hospitalization for COVID-19, if the diagnosis occurs 48 hours after hospitalization for COVID-19, this is defined as a superinfection. (Garcia-Vidal C *et al.*, 2021)

From current study of fungal infections in COVID-19 patients, was found that 32.5% of patients acquired a secondary bacterial infection, while 25.2% had a secondary fungal infection. (Mina S *et al.*, 2022) Another study found fungal co-infection was identified in 7/989 patients (0.7%). Studies from the COVID-19 outbreak, found fungal co-infections in patients with COVID-19, of 54 case reports and 17 case series were identified, and 181 patients infected with COVID-19 and fungal infections were recorded. (Seyedjavadi SS *et al.*, 2021)

A meta-analysis study of 6639 articles screened, 118 articles were included, found that bacterial co-infections 8%, bacterial superinfections 20%, fungal co-infections 4%, and fungal superinfections 8%. (Musuuza JS *et al.*, 2021) Other retrospective cohort study, found that bacterial co-infection in hospitalized COVID-19 patient was lower than the incidence of bacterial or fungal infections in other influenza, such as H1N1 influenza. (Garcia-Vidal C *et al.*, 2021)

This influenza emerged in 2009 and was associated with high mortality rates or H3N2 influenza. (Martin-Loeches I *et al.*, 2017) Another study about incidence of aspergillosis, 5 cases of invasive aspergillosis occurring in severely immunosuppressed patients hospitalized with pandemic influenza A (H1N1), which this influenza may predispose immunocompromised patients to develop invasive aspergillosis. (Garcia-Vidal C *et al.*, 2011) Garcia et al (2021) concluded in their study that the rates of bacterial, fungal, and viral coinfection and superinfection in hospitalized patients with COVID-19 are low, but if present can lead to severe disease with a worse prognosis. (Garcia-Vidal C *et al.*, 2021) Of the many published articles on SARS-CoV-2 with clinical data, only a few reported bacterial and fungal co-infections but many did not identify the pathogen in detail. Meanwhile, widespread, and inappropriate use of antibiotics still occurs so Chen et al (2020) recommend empirical treatment based on the clinical symptoms of coronavirus patients, choosing the most appropriate antibacterial agent according to local guidelines and local drug sensitivity models, and degrade as early as possible based on microbiological results or stop misapplication of antibiotics. (Chen X *et al.*, 2020)

Studies have reported that the viral and bacterial interaction may include several mechanisms, there are the destruction of the respiratory epithelium where SARS-CoV-2 attacks respiratory epithelial cells and damages epithelial layer integrity, upregulation of molecules that bacteria use as receptors, impaired function of immune cells, including neutrophils and macrophages, the latter affected by the release of interferon-gamma produced during T-cell responses to influenza, that impairs clearance of pneumococci for the lung by alveolar macrophages and this can cause mucus accumulation and increase the risk of bacterial infection.(Feldman C & Anderson R, 2021)

Bacterial and fungal Infection in COVID-19 patients involves a complex interaction between the SARS-CoV-2 virus and the body’s compromised immune response. Especially in cells which involved the humoral and cellular immune response, another mechanism could also be due to increase of susceptibility to opportunistic bacterial infections. For secondary infection by fungi can be happen due to excessive antibiotic use, which can disrupt the balance of normal flora in the body and lead to growth of pathogenic fungi. (Langford BJ *et al.*, 2020) COVID-19 infection impairs immune function and damages lung tissue, allowing for secondary infections by bacteria and fungi that otherwise may not have been able to establish infection. Studies have found high rates of secondary bacterial pneumonia and Aspergillus fungal infections in critically ill COVID-19 patients. (Martin-Loeches I *et al.*, 2017)

In this study, of 30 patients with sputum culture results of gram-negative bacteria, we found that *Acinetobacter baumannii* is the most prevalent gram-negative bacteria with 14 patients (46.7%) followed with *Klebsiella pneumonia* in 5 patients (16.1%), *Pseudomonas aeruginosa* in 5 patients (16.1%) and *Escherichia coli* in 3 patients (9.6%). A study conducted at the Hospital in Zanj an (2021), the same results were also found, the most common bacteria was *Acinetobacter baumannii* from sputum culture (15.4%) and blood (2.1%) results. (Moradi N *et al.*, 2021) Garcia-Vidal et al (2021) report their experience of co-infection and superinfection in hospitalized patients with COVID-19, from 31/989 presented with co-infection, majority of these were respiratory bacterial infections with Streptococcus pneumoniae and Staphylococcus aureus pneumonia. (Garcia-Vidal C *et al.*, 2021)

Out of 10 patients with sputum culture result of fungal, frequency distribution of fungal types was found in 5 patient (50%) with the most common growth were *Candida Albicans*, followed by 3 patient (30%) with *Candida Glabrata* and 2 patient (20%) with *Candida Tropicalis*. The incidence of other pathogen infections was also found in China, among the 99 cases, *Aspergillus flavus, Candida glabrata* and *Candida albicans* were the most common fungal co-infection. (Chen X *et al.*, 2020)

According to Mina et al (2022), the co-pathogenesis of viral and fungal co-infections is characterized by complex interactions and involves dynamic interactions between the virulence of co-infected pathogen and the body’s immune defense. So, an inflammatory process occurs which results in physical disorders and dysregulation of immune response. To unravel the complex pathogenesis of fungal infection and COVID-19 requires an understanding of molecular and physiological aspects. (Mina S *et al.*, 2022) There are several potential risks identified, namely the use of immunosuppressive/immunomodulatory therapy, previous use of antibiotics, chronic lung disease, malignancy, use of ventilators and uncontrolled diabetes. (Wu G *et al.*, 2023) Inflammatory response caused by immune regulation during COVID-19 is the main factor driving fungal infections. The need for diagnostics of fungal infections in COVID-19 cases, especially in patients with clinical support, is very important to prevent an increase in the incidence of morbidity and mortality. The need to use of steroids wisely is a crucial step to limit the occurrence of fungal infections in hospitals. (Seyedjavadi SS *et al.*, 2022)

Although the reported incidence of bacterial or fungal infections in COVID-19 patients is still low but there has been an increase in the rate of administration of antibiotics. Rawson et al study in 2010 as 1450 patients (72%) received antibacterial therapy. The increasing use of antibiotics in COVID-19 patients is a concern which can lead to antimicrobial resistance (AMR). In post-COVID-19 patients with structural damage to the lungs, excessive administration of antibiotics can increase the risk of colonization and infection. (Rawson TM *et al.*, 2020)

In this research, based on antibiotic sensitivity results in COVID-19 patients, revealed that amikacin was the most sensitive antibiotic for gram-negative bacteria *Acinetobacter baumannii* (57.1%) and resistant to cefixime (91,79%) and trimethoprim-sulfamethoxazole (89.64%). *Pseudomonas aeruginosa* bacteria was sensitive to amikacin (100%) and gentamicin (100%). *Klebsiella pneumoniae* most effective antibiotics were amikacin (100%), ertapenem (75%), cefepime (75%) and meropenem (75%), ceftazidime, gentamicin, and piperacillin (50%).

A study in Zanj an (2021) that concerning the frequency and pattern of antimicrobial resistance in COVID-19 patients, they found that gram-negative bacteria were highly sensitive to colistin (97.85%) and widely resistant to cefixime (91.79%) and trimethoprim-sulfamethoxazole (89.64%). Gram-positive bacteria were considerably sensitive to vancomycin (68%) and nitrofurantoin (66%). (Moradi N *et al.*, 2021) Another antibiotic sensitivity pattern from a study in India (2022) revealed that colistin (99%), imipenem (78%), and Fosfomycin (95%) were the most effective drugs against the gram-negative isolates while vancomycin (100%), teicoplanin (99%), and doxycycline (71%) were most potent against the gram-positive isolates. From this study the researcher hopes there will be a useful guide in prescribing an appropriate rational antibiotic in COVID-19 cases which should be properly monitored through proper culture, sensitivity testing and strict antibiotic management. (Sahu C *et al.*, 2022)

The pattern of antifungal sensitivity in our study, found in COVID-19 patients were voriconazole, amphotericin B and micafungin which was the most sensitive (100%), followed with flucytosine, fluconazole and caspofungin (57.1%). Early diagnosis of candidemia as well as treatment with appropriate antifungal therapy is very important, and antifungal drugs that have shown some susceptibility to different Candida species include flucytosine, voriconazole, amphotericin B, itraconazole, and cotrimoxazole. Study by Hatzl et al included 132 patients, of whom 75 (57%) received antifungal prophylaxis (98% posaconazole). In ICU patients with COVID-19 ARF, antifungal prophylaxis was associated with significantly reduced CAPA (COVID-19)-associated pulmonary aspergillosis) incidence, but this did not translate into improved survival. (Pruthi HS, 2022)

The increase in mortality and the difficulty of diagnosis and management of COVID-19 are due to other pathogen infections such as bacteria and fungi. Hence it will be necessary for the current guidelines to provide guidance for clinicians for the management and care of patients with COVID-19-associated bacterial and fungal infections. (Wu HY *et al.*, 2023) A recent systematic review found that, overall, the most common antibiotic classes prescribed were fluoroquinolones (20.0%), macrolides (18.9%), β-lactam/β-lactamase inhibitors (15.0%) and cephalosporins (15.0%), and if empiric antibiotic treatment is administered for suspected coinfections, the choice of antimicrobial should be tailored to likely pathogens and resistance patterns based on local and national guidelines. (Peghin M *et al.*, 2022) For candidemia in COVID-19, fluconazole is recommended as the first-line, empirical therapy for non-critically ill patients or those with a low risk of azole-resistant. (Wu HY *et al.*, 2023) Data regarding the incidence of co-infection and superinfection in COVID-19 patients in Indonesia is still relatively low, but each event rate is very influential on the severity of the disease. The results of this study can be used as a basis for selection of antibiotic type in cases of bacterial or fungal co-infection and/or superinfection in COVID-19 patients, which is in accordance with the warning given by WHO that the use of antibiotics is unnecessary or excessive use can reduce the effectiveness of the drug and trigger bacterial resistance. WHO then recommends that giving antibiotics only be given to COVID-19 patients with suspected co-infection and superinfection and in cases of severe and critical suspicion. (WHO, 2019)

The limitation of this study is the absence of data examined atypical bacteria such as *Mycoplasma*, *Legionella* and *Chlamydia*. Then the second limitation is that it is not described when the examination of sputum samples was carried out and not all subject examined the culture sputum the first day patient hospitalized, so it is not known whether the presence of bacterial and fungal infections is co-infection or superinfection.

## Conclusion

This study shows that there are other pathogenic infections in COVID-19 patients. The most prevalent infections are bacterial infections. Our findings support the need for testing that support the diagnosis of other pathogen infections in COVID-19 patients, such as sputum culture tests. Our findings also provide an overview of antibiotics of what pathogens are obtained in COVID-19 patients, which showed different results for each pathogen, as well as antibiotic sensitivity which can be used as a basis for antibiotic selection for COVID-19 patients.

### Conflicts of interest

All the authors declare that there is no conflict of interest associated with this study.

**List of Abbreviation:**
COVID-19: Coronavirus Disease 2019,
WHO: World Health Organization,
SARS-CoV-2: Severe Acute Respiratory Syndrome Coronavirus 2,
RT-PCR: Reverse transcription polymerase chain reaction,
RNA: Ribonucleic acid,
DNA: Deoxyribonucleic acid,
cDNA: complementary DNA,
ARF: Acute Respiratory Failure,
CAPA: COVID-19-Associated Pulmonary Aspergillosis
